# Early events triggering delayed vasoconstrictor receptor upregulation and cerebral ischemia after subarachnoid hemorrhage

**DOI:** 10.1186/1471-2202-14-34

**Published:** 2013-03-15

**Authors:** Gro Klitgaard Povlsen, Sara Ellinor Johansson, Carl Christian Larsen, Ajoy Kumar Samraj, Lars Edvinsson

**Affiliations:** 1 Department of Clinical Experimental Research, Glostrup Research Institute, Glostrup University Hospital, Nordre Ringvej 69, Glostrup, DK 2600, Denmark; 2 Department of Neurosurgery, Glostrup University Hospital, Glostrup, Denmark

**Keywords:** Cerebral blood flow, Endothelin receptor, 5-Hydroxytryptamine receptor, Neurological outcome, Subarachnoid hemorrhage

## Abstract

**Background:**

Upregulation of vasoconstrictor receptors in cerebral arteries, including endothelin B (ET_B_) and 5-hydroxytryptamine 1B (5-HT_1B_) receptors, has been suggested to contribute to delayed cerebral ischemia, a feared complication after subarachnoid hemorrhage (SAH). This receptor upregulation has been shown to be mediated by intracellular signalling via the mitogen activated protein kinase kinase (MEK1/2) - extracellular regulated kinase 1/2 (ERK1/2) pathway. However, it is not known what event(s) that trigger MEK-ERK1/2 activation and vasoconstrictor receptor upregulation after SAH.

We hypothesise that the drop in cerebral blood flow (CBF) and wall tension experienced by cerebral arteries in acute SAH is a key triggering event. We here investigate the importance of the duration of this acute CBF drop in a rat SAH model in which a fixed amount of blood is injected into the prechiasmatic cistern either at a high rate resulting in a short acute CBF drop or at a slower rate resulting in a prolonged acute CBF drop.

**Results:**

We demonstrate that the duration of the acute CBF drop is determining for a) degree of early ERK1/2 activation in cerebral arteries, b) delayed upregulation of vasoconstrictor receptors in cerebral arteries and c) delayed CBF reduction, neurological deficits and mortality. Moreover, treatment with an inhibitor of MEK-ERK1/2 signalling during an early time window from 6 to 24 h after SAH was sufficient to completely prevent delayed vasoconstrictor receptor upregulation and improve neurological outcome several days after the SAH.

**Conclusions:**

Our findings suggest a series of events where 1) the acute CBF drop triggers early MEK-ERK1/2 activation, which 2) triggers the transcriptional upregulation of vasoconstrictor receptors in cerebral arteries during the following days, where 3) the resulting enhanced cerebrovascular contractility contribute to delayed cerebral ischemia.

## Background

Subarachnoid hemorrhage (SAH) after rupture of an arterial aneurysm is associated with high levels of morbidity and mortality. Cerebral ischemia associated with clinical SAH often shows a biphasic course, with an acute drop in cerebral blood flow (CBF) during and immediately after the bleeding and a phase of delayed cerebral ischemia beginning at day 2-4 post-SAH and lasting for up to 14 days in man [1]. This delayed phase is associated with pathological constriction of cerebral arteries (known as cerebral vasospasm, CVS [2-4]).

Many suggestions as to the molecular mechanisms and pathogenic factors behind CVS and delayed cerebral ischemia after SAH have been put forward, including superoxide radical generation induced by the extravasated blood [1], inflammation in the brain and the cerebral vasculature [5], reduced levels of endothelial vasorelaxant factors and elevated levels of vasoconstrictor substances, such as endothelin-1 (ET-1) and 5-hydroxytryptamine (5-HT) [6,7]. The amount of blood in the subarachnoid space after SAH has been shown to correlate with the degree of CVS [8], fibrinolysis of cisternal blood clots have been shown to prevent symptomatic CVS [9], and thus blood cells have for decades been a primary suspected cause of CVS and delayed cerebral ischemia [10]. However, novel data suggest that in addition, the initial rise in intracranial pressure (ICP) and the associated acute reduction in CBF are of crucial importance [11]. Thus, in a rat model of SAH it was shown that if blood was injected prechiasmatically at low pressure (yielding no acute ICP rise and CBF drop), there was no change in CBF and neurology score at two days after SAH, whereas injections of either blood or saline at high pressure (yielding a dramatic acute rise in ICP and drop in CBF) both resulted in significantly reduced CBF and neurology score at two days post-SAH [11]. Accordingly, other studies in a cerebral artery puncture model of SAH have shown that the duration of the acute drop in CBF in the first hour after SAH is a major determinant of mortality and delayed neuronal cell death occurring several days later [12,13]. The emerging picture is that the initial events during and immediately after SAH trigger cellular and molecular responses that later lead to delayed cerebral ischemia and thereby the early events determine the severity of this feared secondary complication of SAH [12,14].

In recent years, a series of studies have revealed a novel aspect of the cerebrovascular pathology associated with delayed cerebral ischemia after SAH, namely expressional upregulation of vasoconstrictor receptors in cerebral arteries. The expression of contractile endothelin B (ET_B_) and 5-hydroxytryptamine 1B (5-HT_1B_) receptors in cerebrovascular smooth muscle cells is increased two days post-SAH, increasing the contractile reactivity of cerebral arteries towards agonists of these receptors [15,16]. The upregulation of these receptors has been shown to be mediated by intracellular signalling via the mitogen activated protein kinase kinase (MEK1/2) - extracellular regulated kinase 1/2 (ERK1/2) pathway [17,18]. However, it is not known what event(s), acute or more delayed, that trigger the process of vasoconstrictor receptor upregulation, and the timing of the involvement of the MEK-ERK1/2 signalling pathway in the process has not been addressed either.

We hypothesise that the drop in blood flow and wall tension experienced by cerebral arteries in acute SAH is a key triggering event, which via early MEK-ERK1/2 activation in cerebral arteries initiates the process of delayed cerebrovascular vasoconstrictor receptor upregulation contributing to delayed cerebral ischemia. The aim of the present study is to investigate whether the duration of the acute CBF drop during SAH determines the degree of early MEK-ERK1/2 signalling in cerebral arteries, delayed vasoconstrictor receptor upregulation and delayed cerebral ischemia. Also, we aimed to investigate whether inhibition of the MEK-ERK1/2 pathway in an early time-window after SAH would prevent delayed vasoconstrictor receptor upregulation and neurological deficits. We use a rat SAH model in which a fixed amount of blood is injected into the prechiasmatic cistern either at a high rate resulting in a short acute CBF drop or at a slower rate resulting in a prolonged acute CBF drop. We show that a prolonged acute CBF drop triggers early MEK-ERK1/2 activation in cerebral arteries that again is a key triggering event for delayed vasoconstrictor receptor upregulation and cerebral ischemia.

## Methods

### Rat subarachnoid hemorrhage model

All procedures were performed strictly within national laws and guidelines and were approved by the Danish Animal Experimentation Inspectorate (license no. 2011/561-2025).

SAH was induced as described in detail before [19], except for the variation that in the present study the prechiasmatic blood injection was performed at different rates to induce short and prolonged acute CBF drops, as described below. Male Sprague-Dawley rats were anesthetized using 3.5% Isofluran (Abbott Laboratories, Illinois USA) in atmospheric air/O_2_ (70%:30%). Rats were orally intubated and artificially ventilated with inhalation of 1-2% Isofluran in N_2_O/O_2_ (70%:30%) during surgery. Blood samples were regularly analysed in a blood gas analyser (Radiometer, Denmark). Body temperature was kept at 37°C ± 0.5°C with a regulated heating pad. Mean arterial blood pressure (MABP) and ICP were continuously measured via catheters inserted into the tail artery and the cisterna magna, respectively, connected to pressure transducers and a Powerlab and recorded by the LabChart software (all from AD Instruments, Oxford, UK). A laser-Doppler blood flow meter probe was placed on the dura through a hole in the skull drilled 4 mm anterior from bregma and 3 mm rightwards of the midline. Through a second hole drilled 6.5 mm anterior to bregma in the midline, a 27G blunt cannula was descended stereotactically at an angle of 30° to the vertical plane towards a final position of the tip immediate anteriorly to the chiasma opticum. After 30 minutes of equilibration, 250 μl of blood was withdrawn from the tail catheter and injected manually through the cannula.

The pressure and rate of the blood injections was carefully controlled (manually) aiming at raising ICP to the higher range of mean MABP levels in all animals (app. 120 mmHg). At the same time, the injection rate was controlled in order to produce either a short acute CBF drop (relatively fast blood injections) or a prolonged acute CBF drop (relatively slow injections). This was done by following the ICP increase closely on the monitor while adjusting the rate and pressure of the blood injection until the intended ICP peak is reached. Due to variability between rats in the pressure and rate of injection needed to raise ICP to this level, the injection could be sustained for variable periods of time after reaching the ICP peak, thus giving rise to shorter or longer acute CBF drops.

Subsequently, rats were maintained under anaesthesia for another 60 minutes while continuing ICP and CBF recordings. At the end of the procedure, the ICP catheter was cut and sealed 0.5 cm from the tip. However, in rats to be treated with U0126 or vehicle, the ICP catheter was cut 2 cm from the tip and closed with a removable plug in order to be used for later treatment administration. The tail catheter, needle and laser-Doppler probe were removed and incisions closed. Rats were revitalized and extubated. At the end of surgery and every 24 hours thereafter rats received subcutaneous injections of Carprofen (4 mg/kg) (Pfizer, Denmark) and 15 ml isotonic saline. Carprofen is a non-steroidal anti-inflammatory analgesic drug used here due to its long-lasting analgesic effect. We have earlier demonstrated that the employed dose of Carprofen does not prevent SAH-induced vascular inflammation [20,21], an important aspect of the cerebrovascular pathology after SAH. Sham-operated rats went through the same procedure with the exception that no blood was injected intracisternally.

### Treatment and experimental groups

65 untreated rats were operated for this study; 32 rats (20 SAH and 12 sham) in the 3 days group (terminated at 3 days post-SAH/sham surgery), 15 rats (all SAH) in the 4 days group (of which 10 were terminated at day 4 post-SAH and 5 died between day 3 and 4 post-SAH) and 18 rats (9 SAH and 9 sham) in the early time-point groups (terminated either 1 h or 6 h post-SAH/sham surgery). Animals were randomly selected for sham-operation or SAH-induction, and SAH rats were randomly selected for induction of short or long acute CBF drops.

**Figure 1:**
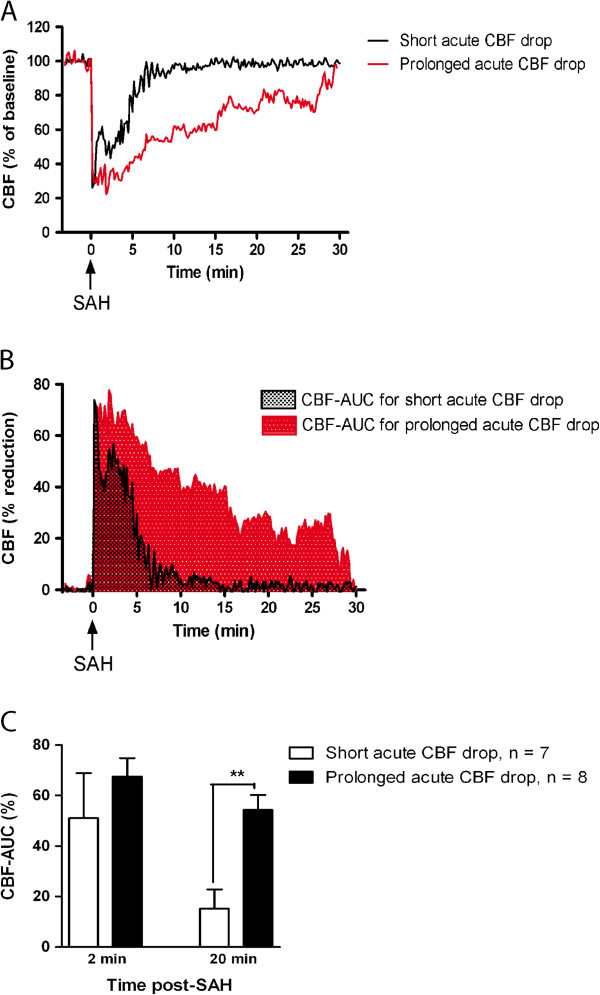
**Short and prolonged acute CBF drops during SAH. **(**A**) Representative laser-Doppler CBF measurements obtained during the first 30 min after induction of SAH. Curves are typical examples of the course of a short acute CBF drop (black curve) and a prolonged acute CBF drop (red curve). (**B**) Same representative laser-Doppler measurements as in (**A**), but depicted as the reduction in CBF as % of baseline values. Shown are typical examples of area under the curve (CBF-AUC) for a short acute CBF drop (grey area) and a prolonged acute CBF drop (red area). (**C**) Bar graph showing mean CBF-AUC values for the first 2 minutes after SAH (CBF-AUC_2 min_) and the first 20 minutes after SAH (CBF-AUC_20 min_) for SAH rats with short and prolonged acute CBF drops, respectively. Error bars are all s.e.m. **: p < 0.01 as determined by student’s t-test.

As illustrated in Figure 1, CBF recordings from the first hour after SAH were transformed to curves of CBF reduction as percentage of baseline values, and the integrals (area under the curve) of these curves were calculated over different time intervals (2 min and 20 min post-SAH, termed CBF_2 min_ and CBF_20 min_, respectively). Based on these values, the SAH rats were divided into two subgroups with CBF_20 min_ below and above 40%, respectively. These subgroups are designated ‘short acute CBF drop’ and ‘prolonged acute CBF drop’, respectively. The choice of the 40% CBF-AUC cut-off value was based on pilot experiments indicating that significant delayed cerebral vasoconstrictor receptor upregulation after SAH was only observed in animals with CBF-AUC_20 min_ values above 40%.

In addition, 14 SAH rats were operated for treatment with either U0126, a specific MEK1/2 inhibitor, or its vehicle (8 vehicle-treated and 6 U0126-treated). Only SAH rats with prolonged acute CBF drops were used for these experiments. Animals were randomly selected for vehicle or U0126 treatment. Animals in the U0126 groups were treated with 0.05 ml/kg body weight of a 10^-5^ M solution of U0126 ethanolate (Sigma-Aldrich, St. Louis, MO, USA) (yielding a final dose of 0.37 μg / kg body weight) diluted in isotonic saline plus 0.1% DMSO. Animals in the vehicle group were treated with 0.05 ml/kg body weight isotonic saline plus 0.1% DMSO. Treatment was administered at 6, 12, and 24 h post-SAH intracisternally through the ICP catheter in the cisterna magna. Animals were then left untreated until termination at 72 h post-SAH.

### Rotating pole test

Gross sensorimotor function was evaluated as the ability of the animals to balance and coordinate their movements when traversing a horizontal pole, which can be either steady or rotating (3 rpm) [22]. At one end of the pole (45 mm in diameter and 150 cm in length) a cage with bedding material from the home cage of the rat being tested and with an entrance hole facing the pole was placed. Performance of the rats was scored according to the following definitions: Score 1) Unable to balance on the pole and falls off immediately, score 2) Balances on the pole but has severe difficulty crossing the pole and moves < 30 cm, score 3) Embraces the pole with paws and does not reach the end of the pole but does manage to move > 30 cm, score 4) Traverses the pole but embraces the pole with paws and/or jumps with hind legs, score 5) Traverses the pole with normal posture but with > 3 foot slips, score 6) Traverses the pole perfectly with < 3 foot slips. On the day before surgery all animals were trained until they obtained score 5-6. On each day after SAH, animals were tested twice on the static pole, once with rotation to the left and once with rotation to the right. The subsequent tests were performed by personnel blinded as regards experimental groups of the animals and always performed in the morning to minimise diurnal rhythm variation.

### [^14^C] iodoantipyrine method for measurement of CBF

Global cortical CBF was measured by the [^14^C] iodoantipyrine method originally described for autoradiographic measurements of CBF [23] and later modified for direct scintillation on brain tissue [24]. In brief, rats were anesthetised with 3.5% Isofluran in atmospheric air/O_2_ (70%:30%), intubated and kept artificially ventilated and anesthetised with 1-2% Isofluran in N_2_O/O_2_ (70%:30%). Respiration was regulated according to regular analyses of blood gases, and body temperature was kept at 37°C ± 0.5°C with a regulated heating pad. MABP was continuously measured via a femoral artery catheter, and a catheter for heparin injection and ^14^C-iodoantipyrine 4[N-methyl-^14^C] infusion was inserted into a femoral vein. After 30 min of equilibration, a bolus injection of 20 μCi ^14^C-iodoantipyrine 4[N-methyl-^14^C] (Perkin-Elmer, Boston, USA) in saline was given (i.v.). At the start of the isotope injection and for the following 24 seconds, one drop of arterial blood was sampled every two seconds. At 24 seconds after isotope injection, rats were decapitated and the brains removed. Cerebellum and brain stem were removed and the cortex from both hemispheres was cleared of subcortical white matter and meninges with associated larger vessels. Within 1 min after decapitation, the cortex tissue was cut in smaller pieces, transferred to scintillation vials and weighed. Tissue samples weighed 100 ±12 mg. Samples were dissolved in 1 ml BTS-450 (Beckman Coulter, CA, USA) for every 100 mg tissue, and digested at 60°C for 3 hours. Samples were then decolorised with 0.4 ml 30% H_2_O_2_ for 1 hour and chemiluminiscence was eliminated by addition of 70 μl glacial acetic acid to each sample. After addition of 10 ml Ready Organic scintillation liquid (Beckman Coulter), vials were counted in a Beckman Liquid Scintillation Counter (Beckman Coulter).

Arterial blood samples were transferred to scintillation vials containing 1 ml of a 1:1 mixture of Soluene-350 (Perkin-Elmer, MA, USA) and isopropanol and dissolved for 2 h at +60°C. Samples were decolorised with 0.2 ml 30% H_2_O_2_ for 30 min at room temperature and then heated to 60°C for 30 min. 10 ml Ready Organic scintillation liquid was added and vials were counted as above. CBF was calculated by solving the equation [23,24].

$$C_{i}\left(T\right)=f\times \text{exp}\left(-\mathrm{kT}\right)\int_{0}^{T}C_{a}\left(t\right)\text{exp}\left(\mathrm{kt}\right)\mathrm{dt}$$

for f, representing CBF. T denoted the time at decapitation, i.e. 24 sec; C_i_(T) the ^14^C-iodoantipyrine content per unit weight of brain tissue at time T; C_a_(t) the arterial concentration of ^14^C-iodoantipyrine at time t; and k = f/λ the rate constant, where λ = 0.78 is the partition coefficient between blood and brain at equilibrium.

### Harvest of cerebral arteries

After decapitation, brains were removed and chilled in cold bicarbonate buffer solution before isolation of middle cerebral arteries (MCA) and basilar arteries (BA) by dissection.

### *In vitro* pharmacology

A wire myograph (Danish Myograph Technology A/S, Aarhus, Denmark) was used to record isometric tension in segments of isolated BA [25]. One mm long vessel segments were mounted in the myograph and immersed in a 37°C running buffer solution of the following composition (mmol/L): NaCl 119, NaHCO_3_ 15, KCl 4.6, MgCl_2_ 1.2, NaH_2_PO_4_ 1.2, CaCl_2_ 1.5 and glucose 5.5. The buffer was continuously aerated with 5% CO_2_ maintaining a pH of 7.4. The vessel segments were stretched to an initial pretension of 2 mN/mm and allowed to equilibrate at this tension for 30 min. The vessels were then exposed to a solution of 63.5 mM K^+^ obtained by partial substitution of NaCl for KCl in the above described buffer. The K^+^-induced contractile responses were used as reference values for normalisation of agonist-induced responses. Only BA with K^+^-induced responses over 2 mN were used for experiments. Concentration-response curves were obtained by cumulative application of 5-carboxamidotryptamine (5-CT) (Sigma, St Louis, MO, USA) in the concentration range 10^-12^ to 10^-4^ M and ET-1 (AnaSpec, San Jose, CA, USA) in the concentration range 10^-14^ to 10^-7^ M. The presence of functional endothelium in the vessel segments was assessed by means of precontraction with 5-HT (3 × 10^-7^ M) followed by relaxation with carbachol (10^-5^ M) as described in ^17^. A relaxant response to carbachol was considered indicative of a functional endothelium, and only vessels showing a relaxant response to carbachol of at least 20% of the precontracted tension were used for further experimentation.

### Immunohistochemistry

4 mm long MCA segments were imbedded in Tissue-Tek (Gibco, Invitrogen A/S, Taastrup, Denmark) and frozen on dry ice. 10 μm thick sections were prepared in a cryostat (Leica Microsystems GmBH). After fixation in Stephanini’s fixative the sections were pre-incubated with phosphate-buffered solution (PBS) containing 5% donkey serum (Jackson ImmunoResearch Europe) and 1% bovine serum albumin (BSA). The primary antibodies used were sheep anti-ET_B_ (Alexis Biochemicals) diluted 1:250, rabbit anti-5-HT_1B_ (Abcam) diluted 1:200 and mouse anti-β-actin (Abcam) diluted 1:500. Secondary antibodies used were DyLight 488-conjugated donkey anti-sheep antibody diluted 1:200, DyLight 488-conjugated donkey anti-rabbit antibody 1:200 and DyLight 549-conjugated donkey anti-mouse antibody 1:200 (all from Jackson ImmunoResearch Europe). All antibodies were diluted in PBS containing 1% BSA, 0.25% Triton X-100 and in addition, primary antibody dilution buffer contained 2% donkey serum. On negative control slides, primary antibodies were omitted. Secondary antibodies were detected at appropriate laser wavelengths in a confocal microscope (Nikon D-eclipse C1, Nikon Instruments).

### Calculations and statistics

Data are presented as means ± standard error of the mean (s.e.m.), n refers to the number of rats. Statistical analyses of rotating pole and CBF data were performed using one-way ANOVA or student’s t-test as indicated in the figure legends. CBF and immunohistochemistry data were analysed using one-way ANOVA followed by Bonferroni’s posttest. Concentration-contractility curves were analysed using two-way ANOVA.

## Results

### Subarachnoid hemorrhage model

In all rats, MABP (98 ± 11 mmHg), PaO_2_ (18 ± 2.1 kPa), PaCO_2_ (4.2 ± 1.4 kPa), pH (7.4 ± 0.7), and temperature were within acceptable physiological limits during surgery, and there were no differences in physiological parameters between groups. The acute mortality rate during the first 24 hours after surgery was 9% for all SAH animals and 3% for all sham-operated animals. There was no significant difference in mortality between vehicle and U0126 treatment groups (all terminated at day 3 post-SAH). After day 3 post-SAH, a considerable delayed mortality was observed: Out of 15 rats in the 4 days SAH group surviving the acute mortality, 5 rats (33.3%) died during day 3 after SAH, whereas 10 survived until day 4 where they were terminated. In sham-operated rats no delayed mortality was observed.

As a result of injecting blood prechiasmatically, ICP increased from 8 ±2 mmHg to 168 ±38 mmHg and cortical CBF dropped to 13 ± 8% of resting flow (average values for all SAH animals).

Two different groups of SAH animals were produced, differing in the duration of the acute CBF drop (see Figure 1A) but with the same amount of blood injected. In one group, the prechiasmatic blood was injected with a relatively high velocity yielding a short acute CBF drop, whereas in the other group the blood was injected at a somewhat slower rate, yielding a more prolonged acute CBF drop. The final division of SAH rats into these two groups was based on integral values (areas under the curve) for time vs. CBF-reduction curves (see Figure 1B) for the first 20 minutes after SAH, termed CBF-AUC_20 min_:

$$\text{CBF}-\mathrm{AU}C_{20\;\text{min}}=\int_{0}^{20}\text{\%CBFreduction}\left(t\right)\mathrm{dt}$$

The SAH subgroup with short acute CBF drops consisted of rats with CBF-AUC_20 min_ < 40%, whereas the group with prolonged acute CBF reduction consisted of rats with CBF-AUC_20 min_ > 40%. This cut-off value was chosen based on pilot experiments indicating that significant delayed cerebral vasoconstrictor receptor upregulation after SAH was only observed in animals with CBF-AUC_20 min_ values above 40%.

As shown in Figure 1C, there was no significant difference (95% confidence interval for the difference was -57, 25 to 24,18) between these subgroups in CBF-AUC values the first 2 min after blood injection, which is the time interval in which the ICP was strongly increased. Moreover, there was no difference in the course of the acute ICP rise between the subgroups (ICP returned to less than 20% of the peak value within 5 minutes after injection of blood and was normalised to baseline values after 15 minutes in all animals). Thus, the differences in CBF-AUC_20 min_ values were not associated with differences in initial ICP rise or CBF drop the first 2 min post-SAH.

### Duration of the acute CBF drop determines delayed CBF reduction, neurological deficits and mortality

The here employed rat SAH model, in similarity with clinical SAH, implies two phases of reduced CBF; an acute CBF drop induced by the prechiasmatic injection of blood followed by a period with normal CBF (in this study, CBF values were normalised to baseline values within the 1h post-SAH recording period in all rats, also rats with prolonged acute CBF drops), then a secondary phase of reduced CBF commencing around 24 h post-SAH and lasting for several days [26,27]. Correlating in time with the second phase of CBF reduction, the animals develop neurological deficits evident as decreased performance in a rotating pole sensorimotor test (see also [18]) and at the same time a considerable delayed mortality (33%) is observed among the SAH rats.

**Figure 2:**
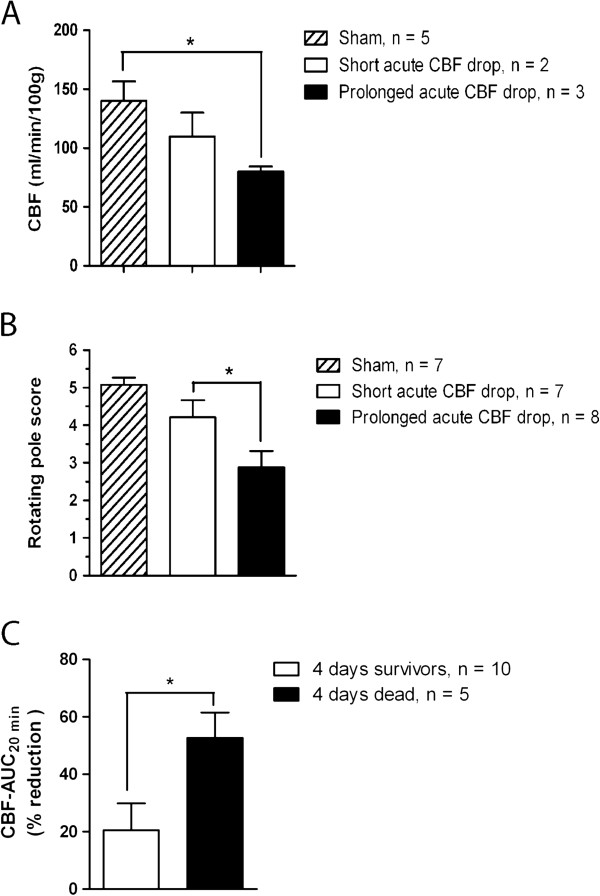
**Duration of acute CBF drop during SAH determines delayed neurological deficits, CBF reduction and mortality. **(**A**) CBF measured by the ^14^C-iodoantipyrine method at 3 days after surgery in sham-operated rats, SAH-induced rats with prolonged acute CBF drop and SAH-induced rats with short acute CBF drop. (**B**) Rotating pole scores obtained at 3 days after surgery by sham-operated rats, SAH-induced rats with short acute CBF drop and SAH rats with prolonged acute CBF drop. (**C**) CBF-AUC_20 min_ values for animals surviving until termination at 4 days post-SAH (4 days survivors) and animals that died at 3-4 days post-SAH (4 days dead), respectively. All data are means ± s.e.m. *: p < 0.05, **: p < 0.01 as determined by student’s t-test.

To investigate the importance of the duration of the acute CBF drop for development of delayed CBF reduction and neurological deficits, we assessed these end-points at 3 days post-SAH in rats with short and prolonged acute CBF drops, respectively. We found that rats with prolonged acute CBF drops displayed somewhat stronger CBF reduction (Figure 2A) and significantly stronger neurological deficits (Figure 2B) than rats with short acute CBF drops. Moreover, to investigate the importance of the acute CBF drop duration for the delayed mortality observed after day 3 post-SAH, we compared CBF-AUC_20 min_ values of rats that survived beyond day 3 post-SAH (66% of the group) and rats that died during day 3 post-SAH (33% of the group). We found that all animals dying in the delayed mortality phase had CBF-AUC_20 min_ values > 40% whereas animals surviving until day 4 after SAH all had CBF-AUC_20 min_ values < 40% (Figure 2C).

### Duration of the acute CBF drop determines the degree of delayed upregulation of ET_B_ and 5-HT_1B_ contractile receptors in cerebral arteries

It has earlier been demonstrated that cerebral arteries increase their expression of contractile ET_B_ and 5-HT_1B_ receptors at 24-48 h post-SAH, and this enhanced expression is associated with enhanced contractile responses to agonists of these receptors [18,27]. It has been hypothesised that this contributes to development of CVS and delayed cerebral ischemia after SAH. To investigate whether also this delayed upregulation and enhanced contractile function of vasoconstrictor receptors is determined by the duration of the acute CBF drop, we compared the function and expression of these receptors in cerebral arteries from SAH rats with short and prolonged acute CBF drops, respectively.

**Figure 3:**
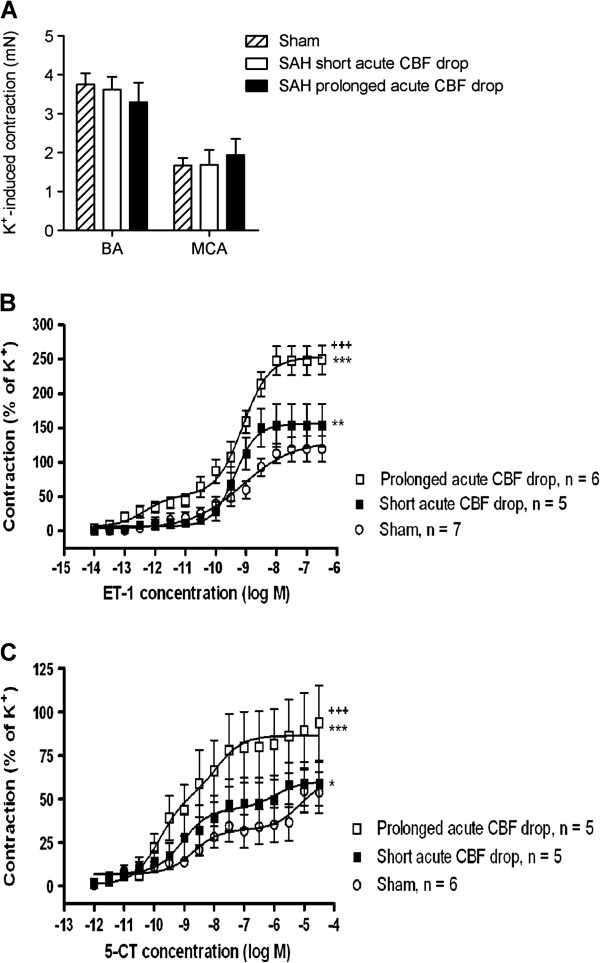
**Duration of acute CBF drop during SAH determines degree of delayed upregulation of contractile receptors in cerebral arteries. **(**A**) Contractile responses induced by 63 mM K^+ ^in middle cerebral arteries (MCA) and basilar arteries (BA) for sham-operated rats and SAH rats with short and prolonged acute CBF drops. Data are mean ± s.e.m. of maximal K^+^-induced contractions for each vessel segment. (**B** and **C**) Concentration-contraction curves for basilar arteries stimulated with cumulative doses of ET-1 (**B**) or 5-CT (**C**). Shown are data obtained at 3 days after surgery in sham-operated rats, SAH-induced rats with short acute CBF drop and SAH-induced rats with prolonged acute CBF drops. Data are mean ± s.e.m as percent of 63 mM K^+^-induced contraction. Stars indicate significant differences between sham and SAH-induced animals as determined by two-way ANOVA (***: p < 0.001). Crosses indicate significant differences between SAH-induced rats with short and prolonged acute CBF drop, respectively, as determined by two-way ANOVA (+++: p < 0.001).

To assess the degree of enhanced contractile function of ET_B_ and 5-HT_1B_ receptors in cerebral arteries we measured contractile responses to the endothelin receptor agonist ET-1 and the 5-HT_1_ receptor agonist 5-CT, respectively. Potassium-induced contractile responses were used as internal controls for normalization of agonist-induced responses. Potassium-induced responses did not differ significantly between experimental groups (Figure 3A).

It has earlier been demonstrated that SAH results in a left-wards shift of ET-1 concentration-contraction curves and a transition into biphasic curves (from sigmoidal curves in sham-operated rats), reflecting the occurrence of contractile ET_B_ receptors in the smooth muscles of cerebral arteries in addition to the contractile ET_A_ receptors already present there [16]. Moreover, it has been shown that SAH results in a leftwards shift of 5-CT concentration-contraction curves and that this shift reflects upregulation of 5-HT_1B_ receptors specifically [15,28]. We here demonstrate that the SAH-induced enhancement of cerebrovascular contractile responses to ET-1 and 5-CT was significantly stronger in SAH rats with prolonged acute CBF drop than with short acute CBF drops (Figure 3B and C). In fact, contractile responses in SAH rats with short acute CBF drops were only slightly stronger than the responses in sham-operated rats (Figure 3B and C).

**Figure 4:**
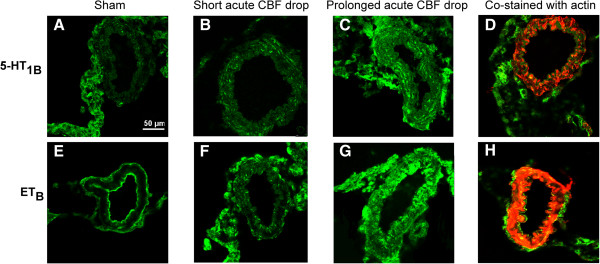
**5-HT**_**1B **_**and ET**_**B **_**receptor expression in cerebral arteries. **5-HT_1B _and ET_B_ receptor expression and localisation was determined in middle cerebral artery sections by means of immunohistochemical stainings and confocal microscopy. Shown are representative photomicrographs for sham-operated rats (**A **and **E**), SAH rats with short acute CBF drop (**B **and **F**) and SAH rats with prolonged acute CBF drop (**C **and **G**). Upper row are photomicrographs showing green fluorescent staining with antibodies against the 5-HT_1B _receptor (**A-C**) and an example of a co-staining with antibodies against the 5-HT_1B _receptor (green fluorescence) and smooth muscle actin (red fluorescence) (**D**). Lower row are photomicrographs showing green fluorescent staining with antibodies against the ET_B_ receptor (**E-G**) and an example of a co-staining with antibodies against the ET_B _receptor (green fluorescence) and smooth muscle actin (red fluorescence) (**H**). Two middle cerebral artery sections from each of 6 sham-operated rats and 8 SAH rats (4 from each acute CBF drop duration groups) were analyzed and one representative image for each group is shown.

In addition, we demonstrate by immunohistochemistry that the expression of ET_B_ and 5-HT_1B_ receptor protein in the smooth muscle layer of cerebral arteries was only clearly increased in SAH rats with prolonged acute CBF drop, whereas arteries from SAH rats with short acute CBF drops showed ET_B_ and 5-HT_1B_ receptor levels comparable to sham-operated rats (Figure 4). These findings indicate that the increased levels of ET_B_ and 5-HT_1B_ receptor expression underlies the enhanced contractile function of these receptors after SAH, although it cannot be ruled out that other mechanisms such as changes in ligand binding affinity or coupling efficiency could also be involved.

### Duration of acute CBF drop determines the degree of ERK1/2 activation in cerebral arteries early after SAH

**Figure 5:**
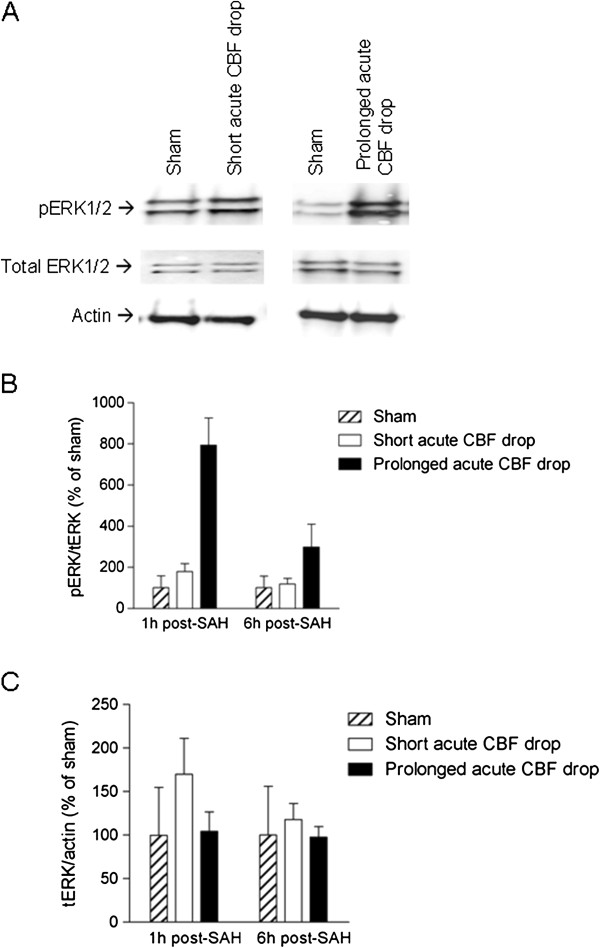
**Duration of acute CBF drop during SAH determines degree of early activation of ERK1/2 in cerebral arteries after SAH. **Immunoblots of phospho-ERK1/2, total ERK1/2 and actin levels in cerebral artery tissue from sham-operated rats, SAH rats with short acute CBF drop and SAH rats with prolonged acute CBF drop. (**A**) Representative immunoblots of cerebral artery tissue from rats terminated 1 h after sham-operation or SAH with short or prolonged acute CBF drop. (**B **and **C**) Band chemiluminiscence intensity quantifications presented as means ± s.e.m. of the ratios between phosphorylated ERK1/2 and total ERK1/2 protein bands (**B**) or ratios between total ERK1/2 and actin protein bands for rats terminated either 1 h or 6 h after SAH or sham-operation. Numbers of rats in the groups are: 1 h Sham n = 5, 1 h SAH short acute CBF drop n = 3, 1 h SAH prolonged acute CBF drop n = 2, 6 h Sham n = 4, 6 h SAH short acute CBF drop n = 2, 6 h SAH prolonged acute CBF drop n = 2.

Activation of the MEK-ERK1/2 signalling pathway has been suggested to trigger upregulation of contractile receptors in cerebral arteries after SAH [17,29]. We therefore investigated the importance of the acute CBF drop duration for activation of this signalling pathway early after SAH. As shown in Figure 5, SAH rats with prolonged acute CBF drop had strongly increased levels of phosphorylated ERK1/2 in cerebral arteries at 1h and at 6h after SAH. In contrast, SAH rats with short acute CBF drops showed only a slightly increased ERK1/2 phosphorylation at 1 h after SAH and no increase in ERK1/2 phosphorylation at 6h after SAH as compared to levels in sham-operated rats. SAH did not change the levels of total ERK expressed in cerebral arteries (Figure 5C). These data suggest that only a prolonged acute CBF drop triggers early ERK1/2 phosphorylation in cerebral arteries after SAH.

### Treatment with a MEK1/2 inhibitor early after SAH prevents delayed upregulation of ET_B_ and 5-HT_1B_ receptors in cerebral arteries and improves neurological outcome

If activation of the MEK-ERK1/2 pathway induced by a prolonged acute CBF drop triggers delayed upregulation of vasoconstrictor receptors in cerebral arteries, is this pathway then acting mainly as a ‘switch-on’ mechanism early after the SAH or is it involved throughout the period of several days post-SAH during which the receptor upregulation process takes place?

**Figure 6:**
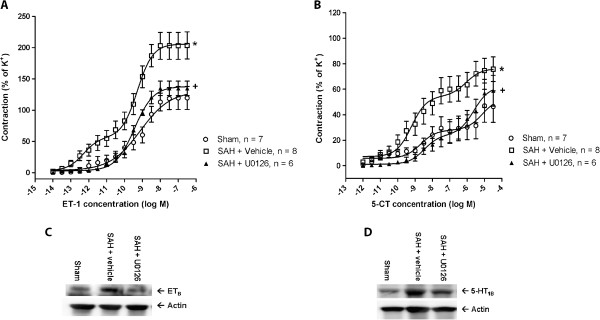
**Early treatment with MEK1/2 inhibitor prevents delayed upregulation of ET**_**B **_**- and 5-HT**_**1B **_**-mediated vasoconstriction and receptor protein expression in cerebral arteries after SAH. **(**A-B**) Concentration-contraction curves obtained at 3 days after sham-operation or SAH for basilar arteries stimulated with cumulative doses of ET-1 (**A**) or 5-CT (**B**). Shown are data for sham-operated rats (Sham), SAH rats treated with U0126 at 6 h, 12 h and 24 h after SAH (SAH + U0126) and SAH rats treated with vehicle at similar time points (SAH + vehicle). All SAH rats included had prolonged acute CBF drops. Data are mean ± s.e.m as percent of 63 mM K^+^-induced contractions. Stars indicate significant differences between sham and SAH-induced animals as determined by two-way ANOVA (*** p < 0.001, ** p < 0.01, * p > 0.05). Crosses indicate significant differences between U0126- and vehicle-treated SAH rats as determined by two-way ANOVA (+++ p < 0.001). (**C-D**): Immunoblots of ET_B _and 5-HT_1B _receptor expression and actin levels (loading control) in cerebral artery tissue from sham-operated rats and SAH rats with prolonged acute CBF drops treated with U0126 or vehicle. The experiment was repeated three times and one representative immunoblot for each receptor type is shown.

To address this question, we performed a treatment study using the specific MEK1/2 inhibitor U0126. Only SAH rats with prolonged acute CBF drops (CBF-AUC_20 min_ values > 40%) were included in these experiments. Animals were treated with U0126 at 6 h, 12 h and 24 h post-SAH followed by a period without treatment until termination of the animals at day 3 post-SAH. As shown in Figure 6, this treatment with U0126 completely prevented the SAH-induced upregulation of contractile responses mediated by ET-1 (Figure 6A) and 5-CT (Figure 6B). Moreover, we showed by immunoblotting that the U0126 treatment prevented the SAH-induced increase in ET_B_ (Figure 6C) and 5-HT_1B_ (Figure 6D) receptor protein expression in cerebral artery tissue at 3 days after SAH (Figure 6C and D). Together, these data indicate that the MEK-ERK1/2 pathway plays a critical role only in initiation of the vasoconstrictor receptor upregulation in the first 24 h post-SAH, after which this pathway is no longer critically involved.

**Figure 7:**
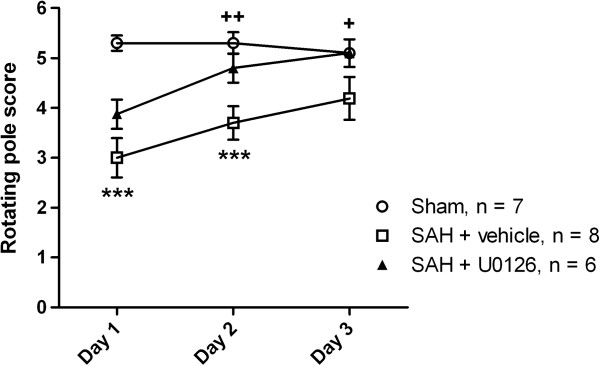
**Early treatment with the MEK1/2 inhibitor U0126 prevents delayed neurological deficits after SAH. **Rotating pole scores obtained at 1, 2 and 3 days after surgery for sham-operated rats and SAH rats with prolonged acute CBF drops treated with U0126 or vehicle. All data are means ± s.e.m. Stars indicate significant differences between sham-operated rats and SAH rats treated with vehicle as determined by one-way ANOVA. ***: p < 0.001. Crosses indicate significant differences between SAH rats treated with vehicle and U0126, respectively, as determined by one-way ANOVA. +: p < 0.05, ++:p < 0.01.

To assess whether inhibition of the MEK-ERK1/2 pathway during the early time-window post-SAH would also improve neurological outcome, we evaluated the neurological function of the rats by means of a rotating pole test. As shown in Figure 7, the U0126 treatment significantly improved neurological function of the rats at day 2 and day 3 post-SAH, at which time point average neurological scores for U0126 treated rats no longer differed from the scores of sham-operated rats, whereas vehicle-treated SAH rats displayed significant neurological deficits at all time points.

## Discussion

This is the first study to demonstrate that the duration of the initial CBF drop induced by injection of a standardised volume of blood into the prechiasmatic cistern is a determinant for a) the degree of ERK1/2 activation in cerebral arteries early after the SAH, b) delayed upregulation of vasoconstrictor receptors in cerebral arteries several days after the SAH and c) delayed CBF reduction, neurological deficits and mortality. Moreover, we show for the first time that treatment with an inhibitor of MEK-ERK1/2 signalling only during an early time window from 6 to 24 h after the SAH is sufficient to completely prevent delayed vasoconstrictor receptor upregulation and improve neurological outcome several days after the SAH. These findings suggest a series of events where 1) the drop in blood flow and wall tension experienced by the cerebral arteries during SAH triggers early activation of the MEK-ERK1/2 pathway, which 2) triggers increased expression and contractile function of vasoconstrictor receptors in cerebral arteries during the following days, where 3) the resulting enhanced cerebrovascular contractility contribute to development of delayed cerebral ischemia evident as CBF reduction, neurological deficits and mortality.

In this study, we investigate the different series of events taking place in two different ‘variants’ of the prechiasmatic injection SAH model differing in the duration of the acute CBF drop which was either short or prolonged. The occurrence of a prolonged acute CBF drop persisting after decline of the initial ICP rise is in accordance with earlier studies showing that acute vasoconstriction takes place after SAH. This can prolong the period of acute CBF reduction beyond the short time interval where ICP is increased to levels above jugular vein pressure [12,13,30,31], a phenomenon that is also thought to take place in clinical acute SAH, at least in some patients. Since other important factors such as the amount of blood injected and the magnitude and duration of the initial ICP increase are kept constant, our experimental set-up reveals pathophysiological events triggered primarily by the acute CBF drop in itself, irrespective of hemorrhage volume and initial ICP increase.

We here demonstrate for the first time in this SAH modality that the duration of the initial CBF drop is a physiological determinant of neurological outcome and mortality during the first 4 days after SAH, a finding which is well in accordance with earlier studies using the endovascular perforation SAH model [12,13]. However, this does not mean that the acute CBF drop is the sole determinant of delayed CBF reduction and delayed cerebral ischemia, and it is important to note that a number of studies have suggested that the amount of blood in the subarachnoid space and the rate of clearance of the blood clot determine the later risk of delayed cerebral ischemia and symptomatic CVS [8,9], and thus the risk of delayed cerebral ischemia appears to be determined by a combination of multiple factors including, but not limited to, the duration of the acute CBF drop.

SAH induced both enhanced contractile function and increased protein expression of ET_B_ and 5-HT_1B_ receptors. We have earlier demonstrated that the increased receptor protein levels are associated with increased receptor mRNA levels [15,16,27], suggesting a transcriptional mechanism of upregulation, however, it cannot be ruled out that other mechanisms, such as reduced mRNA degradation, increased translation efficiency, and decreased receptor turnover, also play a role. We also show for the first time that the degree of cerebrovascular upregulation of ET_B_ and 5-HT_1B_ receptors during the first 3 days post-SAH depends strongly on the duration of the acute CBF drop. This suggests that the lack of flow and wall tension experienced by the cerebral arteries during the initial CBF drop may be the trigger of the receptor upregulation, rather than the exposure to extravascular blood in itself. This conclusion is in accordance with a recent study demonstrating that the degree of upregulation of cerebrovascular ET_B_ and 5-HT_1B_ receptors after transient occlusion of the two common carotid arteries combined with systemic hypotension is strongly dependent on the duration of the carotid artery occlusion [32]. In support for a central role of the drop in vascular wall tension in the initiation of vascular ERK1/2 activation, we have recently shown that in a model of distal MCA occlusion contractile ET_B_ receptors were upregulated only downstream from the occlusion, whereas the immediate upstream MCA, experiencing the same low degree of ischemia in the surrounding tissue but no drop in vascular wall tension, did not show changes in ET_B_ receptor function. Moreover, we have recently demonstrated that the upregulation of contractile ET_B_ receptors taking place during organ culture of cerebral artery segments can be prevented by applying a physiological level of wall tension to the artery segments during organ culture, and that this tension-dependent ET_B_ upregulation is mediated by signalling via the focal adhesion kinase (FAK) known to be associated with integrin mechanosensitive protein complexes at the plasma membrane [33]. These findings point to a vasogenic mechanosensitive trigger of ERK1/2 activation upon drop in wall tension in cerebral arteries, however, it cannot be ruled out that the decreased perfusion could induce the release of an endothelial factor, parenchymal metabolite, glial factor or neurohormone that act on the cerebral arteries to promote increased ERK activation.

The MEK-ERK1/2 signalling pathway has earlier been demonstrated to be involved in the upregulation of cerebrovascular ET_B_ and 5-HT_1B_ receptors after SAH. Thus, inhibition of either MEK1/2 or its upstream activator Raf completely prevents SAH-induced ERK1/2 activation and vasoconstrictor receptor upregulation in cerebral arteries and alleviates delayed cerebral ischemia [17,18,26]. The time-course of ERK1/2 activation in cerebral arteries after SAH was studied in detail in an earlier study, where increased ERK1/2 activity was demonstrated in cerebral arteries at time-points between 1-48 h post-SAH [29]. However, the critical time window during which activation of this pathway drives the upregulation of vasoconstrictor receptors has not hitherto been investigated. Moreover, it has not been investigated whether the activation of the MEK-ERK1/2 pathway in cerebral arteries depends on the duration of the acute CBF drop during SAH. We here demonstrate activation of ERK1/2 in cerebral arteries throughout the first 6h post-SAH only in rats with prolonged acute CBF drops. Moreover, we show that treatment with a MEK1/2 inhibitor from 6 h to 24 h after SAH followed by a two days period without further treatment completely prevents the later enhancement of ET_B_- and 5-HT_1B_-mediated vasoconstriction in cerebral arteries. These findings, together with our demonstration of the importance of the acute CBF drop duration, suggest that the acute CBF drop induces early activation of the MEK-ERK1/2 pathway in cerebral arteries, which then during the time window from 6 to 24 h post-SAH acts as a ‘switch-on’ mechanisms for the expressional and functional upregulation of vasoconstrictor receptors in cerebral arteries over the following couple of days.

A large research effort has been put into findings effective treatments for CVS and delayed cerebral ischemia after SAH. Recently, the CONSCIUOS trials with the ET_A_ receptor antagonist Clazosentan showed that specific targeting of ET_A_ receptors is not sufficient to significantly alleviate delayed cerebral ischemia and improve clinical outcome after SAH [34,35]. One possible explanation for the disappointing clinical effects of ET_A_ receptor inhibition is that the complex vascular pathology after SAH involves many other, perhaps more or equally important, factors such as increased expression of several other vasoconstrictor receptors and their agonists [36], vascular inflammation [5], endothelial apoptosis and blood-brain barrier breakdown [37,38]. The results of the present study underscore the importance of the acute phase of the SAH. We suggest that therapies targeting specific intracellular signal transduction components activated early after the SAH may help prevent the later evolution of SAH-induced vascular pathology contributing to delayed cerebral ischemia. Inhibition of the MEK-ERK1/2 pathway has in other studies been shown to alleviate delayed vascular inflammation, CBF reduction, and neurological deficits after experimental SAH [17,18,20]. The profound effect of MEK1/2 inhibition on vasoconstrictor receptor levels and neurological outcome when administered only from 6 to 24 h post-SAH in the present study, points to this as a possible way of targeting early changes within a clinically realistic therapeutic time window.

## Conclusion

In conclusion, our findings suggest that delayed upregulation of vasoconstrictor receptors in cerebral arteries as well as delayed CBF reduction and neurological deficits several days after an SAH is triggered by the acute CBF drop during the SAH followed by early MEK-ERK1/2 signalling in the cerebral arteries.

## Competing interests

The authors declare that they have no conflicts of interest or financial disclosures.

## Authors’ contributions

GKP performed the majority of the experimental work, designed experiments, analysed data and wrote the manuscript. SEJ and CCL performed parts of the *in vivo* experimental work, AS performed western blots, LE conceived the idea, designed experiments and edited the manuscript. All authors read and approved the final manuscript.
